# Bone protective effect of sinomenine against monosodium iodoacetate induced knee and hip injury in rat model: an inflammatory pathway

**DOI:** 10.1590/acb390924

**Published:** 2024-02-05

**Authors:** Yi-Hao Lei, Xing-Xi Hu, Hong-Jie Wen, Yong-Cheng Deng, Jun-Liang Jiang, Qing-Gang Zhao

**Affiliations:** 1 Affiliated Hospital of Yunnan University, Bone and Traumatic Surgery, Kunming, China.

**Keywords:** Osteoarthritis, Inflammation, Sinomenine, Antioxidants, Matrix Metalloproteinases

## Abstract

**Purpose:**

Osteoarthritis (OA) is a degenerative joint disease which is categorized via destruction of joint cartilage and it also affects the various joints, especially knees and hips. Sinomenine active phytoconstituents isolated from the stem of *Sinomenium acutum* and already proof anti-inflammatory effect against the arthritis model of rodent. In this experimental protocol, we scrutinized the anti-osteoarthritis effect of sinomenine against monosodium iodoacetate (MIA) induced OA in rats.

**Methods:**

MIA (3 mg/50 μL) was used for inducing the OA in the rats, and rats received the oral administration of sinomenine (2.5, 5 and 7.5 mg/kg body weight) up to the end of the experimental study (four weeks). The body and organs weight were estimated. Aggrecan, C-terminal cross-linked telopeptide of type II collagen (CTX-II), glycosaminoglycans (GCGs), monocyte chemoattractant protein-1 (MCP-1), Interferon gamma (IFN-γ), antioxidant, inflammatory cytokines, inflammatory mediators and matrix metalloproteinases (MMP) were analyzed.

**Results:**

Sinomenine significantly (P < 0.001) boosted the body weight and reduced the heart weight, but the weight of spleen and kidney remain unchanged. Sinomenine significantly (P < 0.001) reduced the level of nitric oxide, MCP-1 and improved the level of aggrecan, IFN-γ and GCGs. Sinomenine remarkably upregulated the level of glutathione, superoxide dismutase and suppressed the level of malonaldehyde. It effectually modulated the level of inflammatory cytokines and inflammatory mediators and significantly (P < 0.001) reduced the level of MMPs, like MMP-1, 2, 3, 9 and 13.

**Conclusions:**

Sinomenine is a beneficial active agent for the treatment of OA disease.

## Introduction

The most frequent type of degenerative joint diseases is osteoarthritis (OA). OA is induced via joint cartilage destruction and it affects the entire joints of the body, especially knees and hips^1^^,^ 2. Various risk factors such as obesity, aging, muscle weakness, and injury are involved in the incidence of OA disease^3^. The incidence of the OA upsurges day by day due to increase age^2^. Multiple risk factors for OA disease, such as metabolic processes, inflammation, and molecular and cellular mechanisms, all play a significant role in the disease’s emergence and expansion^2^^,^ 4. Loss of flexibility, swelling, stiffness, tenderness, and pain symptoms are involved in the OA disease. More than 80% of OA patients feel difficulty in routine works. Recently, a published meta-analysis study showed that globally incidence of knee OA was 16% in 15 year-old people and 22.9% in the aged of ≥ 40, with a female to male ratio (1.62)^5^. Due to multiple risk factors involved, it is very difficult to treat the disease.

OA disease differs from the rheumatoid arthritis (RA) disease^3^. The inflammatory response in the joints, as well as the inflammatory reaction involved in joint swelling and pain, is involved in OA disorders^2^^,^ 6. RA is an autoimmune disease and involves the inflammatory response in the expansion of disease^7^. Previous investigation suggested that the enhanced level of cytokines and inflammatory mediators was observed in the synovial fluids of OA patients^6^^,^ 8.

Few studies have suggested that cartilage degradation markers such as C-terminal cross-linked telopeptide of type II collagen (CTX-II) and matrix metalloproteinase (MMP-1 and MMP-3) have a role in the progression and propagation of OA disease^3^^,^ 9. Moreover, the alteration of cartilage degradation marker induces the dysfunction of cartilage matrix related enzymes, which results in cartilage degradation in the joint^10^^,^ 11.

Various factors involved in the expansion of OA disease. MMPs play a crucial role in the degradation of cartilage. MMP role is not restricted to the collage type II, which is made up of 90% cartilage matrix proteins, collage type IV, collage type IX, and proteoglycan^12^. According to a previous study, low-grade joint inflammation plays an important role in the course of OA disease. Nuclear factor-B (NF-κB) is an important mediator of inflammation that has a function in the pathogenesis of OA disease^12^. Recent report suggests that the NF-κB pathway activation increases the level of cytokines, which induces the articular joint destruction, leading the onset and expansion of OA disease^13^^,^ 14. The activation of inflammatory reactions, apoptosis, and MMPs is influenced by oxidative stress and its products. It is also triggered by reactive oxygen species (ROS), which can disrupt and oxidize cartilage homeostasis, while also promoting catabolism by triggering cell death^2^^,^ 15. Due to the role of the inflammatory response in the OA disease, the researcher chooses the inflammatory drug for the treatment of OA disease. Various plant extracts and its phytoconstituents show the pharmacological effect for the treatment of OA rat model.

Currently available treatment for OA disease is nonsteroidal anti-inflammatory drugs like paracetamol (acetaminophen) and aspirin, but both the drugs have limitations due to side effects and effective only relieving the osteoarthritis pain^3^. However, recently intra-articular corticosteroids are recommended for the treatment of OA, but they induce the local and systemic side effects^3^. Furthermore, meta-analysis showed that the various OA supplements such as chondroitin, avocado and glucosamine are used for the OA therapy, but the mechanism is inconclusive^16^.

Due to lack of the effect treatment for the OA disease, the end stage OA condition that time available option is arthroplasty or joint replacement^5^, but arthroplasty is a costly procedure, and there is the chance to develop the infection, periprosthetic fractures, and loosening of prosthetics^17^. Due to the costly procedure, it is urgent more protective drugs with less cost, and no side effects against the OA disease.

Sinomenine is an alkaloid compound isolated from the *Sinomenium acutum* plant native from Japan and China^18^^,^ 19. It exhibits the anti-inflammatory potential against the various immune related diseases in the animal model^20^. Li et al. exhibited the antioxidant and anti-inflammatory effect of sinomenine against gestational diabetes mellitus in female rats^21^. Sinomenine also exhibited anti-inflammatory effect against the complete freund’s adjuvant (CFA) induced chronic inflammatory pain in rats^22^. We investigated the anti-osteoarthritic effect of sinomenine against monosodium iodoacetate (MIA)-induced OA in rats in this experimental study, because of its anti-inflammatory and antioxidant properties.

## Methods

### Animal

Swiss Wistar rats (sex-male; weight 250–280 g; 10–12 weeks old) were obtained from the animal housing and placed in a polyethylene cage (two rats each). The rats were kept in the departmental animal house for adopting in laboratory conditions for one week for acclimatization. The entire animal experiment was conducted in accordance with the institution’s animal protocols. The whole animal study was approved by the institutional ethical committee (approval number: 20210004531).

### Osteoarthritis induction

In order to induce OA in rats, the animals were sedated with isoflurane, and articular cartilage damage was caused using an intra-articular injection of MIA. MIA (3 mg/50 μL) was produced in saline via the right knee’s infrapatellar ligament. The rats were given time to recover from anesthesia and monitored until they resumed normal activity^1^.

### Experimental group

The rats were put into five groups.

- Group I: normal;
- Group II: MIA control;
- Group III: MIA + sinomenine (2.5 mg/kg);
- Group IV: MIA + sinomenine (5 mg/kg);
- Group V: MIA + sinomenine (7.5 mg/kg).

For four weeks, the rats were subjected to the treatment already described. The tested group received a freshly made suspension that was dissolved in carboxymethylcellulose (CMC) and administered to the animal by oral gavage, with the dose of sinomenine chosen based on previously published research^23^. After four weeks, the rats were euthanized. Before being euthanized, the rats were fasted overnight (12 h), and the blood samples of all group rats were collected via cardiac puncturing. The blood samples were centrifuged at 5,000 rpm for 5 min to separate the serum. The serum sample was stored at -20°C. The rats were euthanized at the end of the study, and different organs, like heart, spleen and kidney, were removed and weighted.

### Nitric oxide production

The nitric oxide level was estimated using a previously described method. Griess reaction was used for the determination of nitric oxide. Briefly, serum sample (100 μL) was mixed with the Griess reagent A (50 μL) and equal quantity of Griess reagent B and incubated at room temperature (37°C) for 15 min. Finally, the optical density at 540 nm was estimated.

### Antioxidant parameters

Glutathione (GSH) was estimated using the 5,5-dithiobis (2-nitrobenzoic acid) model, which resulted in a yellow product^24^. The final absorbance was estimated at 405 nm using the commercially available kits (Biodiagnostic kit, Egypt). The level of malonaldehyde (MDA) (MBS7727872) was estimated using enzyme-linked immunosorbent assay (ELISA) kits (MyBioSource Ltd., San Diego, United States of America). The superoxide dismutase (SOD) (MBS8819950) level was estimated using the ELISA kits (MyBioSource Ltd., San Diego, United States of America), following the manufacture’s protocol.

### Enzyme-linked immunosorbent assay

The cartilage degrading marker such as MMP-1 (MBS7720142), 2 (MBS7727654), 3 (MBS7720097), 9 (MBS8124027), 13 (MBS7726087); inflammatory parameters PGE_2_ (MBS7727909), cyclooxygenase-2 (MBS7721379), inducible Nitric oxide synthase (MBS9460915), CTX-II (MBS2533577), interferon gamma (IFN-γ) (MBS697344), monocyte chemoattractant protein-1 (MCP-1) (MBS7720303), glycosaminoglycans (GAGs) (MBS730728), aggrecan (MBS262707), and cytokines like tumor necrosis factor (TNF)-α (MBS508481), interleukin (IL)-1β (MBS697369) and IL-6 (MBS8123859) were estimated according the manufacture’s protocol (MyBioSource Ltd., San Diego, United States of America).

### Statistical analysis

GraphPad Prism 7 software (GraphPad Prism, St. Louis, United States of America) was used for the statistical analysis. The data in this study was given as mean standard error means (SEM) for all the variables. Multiple comparisons were made using one-way and two-way analysis of variance. P was chosen as the significant number.

## Result

### Body and organ weight

**Figure 1:**
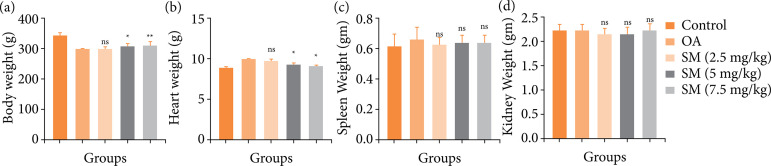
The effect of SM on the body and organ weight of MIA induced OA group rats. **(a)** body weight, **(b)** heart weight, **(c)** spleen weight and **(d)** kidney weight. All the results are presented as mean ± SD (n = 10 for each group) from three independent experiments.

OA: osteoarthritis; SM: sinomenine; MIA: monosodium iodoacetate; SD: standard deviation; *P < 0.05; **P < 0.01; ***P < 0.001 vs. MIA-induced OA group; ^###^P < 0.001 vs. normal control.

During the OA, disease suppressed the body weight. OA disease group rats displayed the reduced body weight due to expansion of the disease, and sinomenine treated group rats exhibited the improvement of the body weight. Sinomenine (7.5 mg/kg) treated rats revealed the maximum enhancement of the body weight. It reached the body weight almost near to the normal group rats (Fig. 1a).

OA control group rats showed the increased heart weight, which was significantly (P < 0.001) repressed by the sinomenine treatment. Sinomenine (7.5 mg/kg) treated group rats demonstrated the heart weight almost near the normal group rats (Fig. 1b). We also estimated the spleen and kidney weight in all group rats. The weight of the spleen (Fig. 1c) and kidney (Fig. 1d) in all groups of rats did not differ statistically.

### Nitric oxide

**Figure 2:**
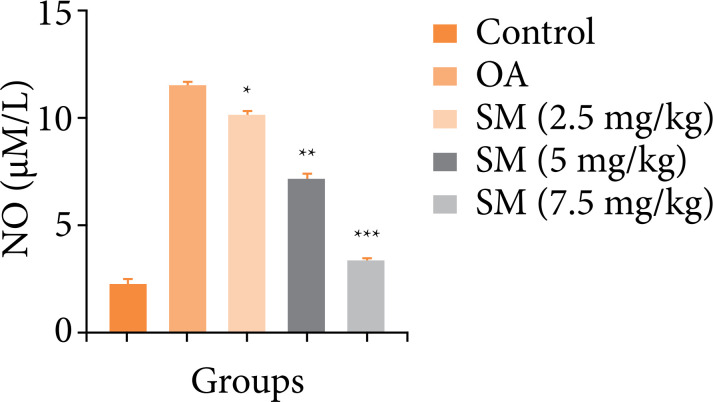
Effect of SM on the nitric oxide of MIA induced OA group rats. All the results are presented as mean ± SD (n = 10 for each group) from three independent experiments.

OA: osteoarthritis; SM: sinomenine; NO: nitric oxide; MIA: monosodium iodoacetate; SD: standard deviation; *P < 0.05; **P < 0.01; ***P < 0.001 vs. MIA-induced OA group; ^###^P < 0.001 vs. normal control.

On the comparison between the groups, OA group rats showed the boosted level of nitric oxide, which was reduced by the sinomenine treatment. Sinomenine treated group rats significantly (P < 0.001) suppressed the level of nitric oxide, and maximum reduction was observed in the sinomenine (7.5 mg/kg) treated group rats (Fig. 2).

### Aggrecan and glycosaminoglycans

**Figure 3:**
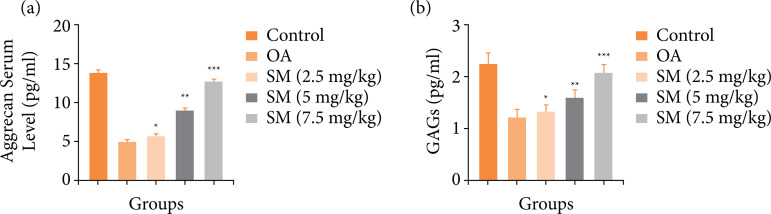
Effect of SN on the aggrecan and GAGs of MIA induced OA group rats. **(a)** aggrecan and **(b)** GAGs. All the results are presented as mean ± SD (n = 10 for each group) from three independent experiments.

OA: osteoarthritis; SM: sinomenine; GAG: glycosaminoglycan; MIA: monosodium iodoacetate; SD: standard deviation; *P < 0.05; **P < 0.01; ***P < 0.001 vs. MIA-induced OA group; ^###^P < 0.001 vs. normal control

The levels of aggrecan and GAGs in the various groups of rats are shown in Fig. 3. OA-induced group rats had lower levels of aggrecan (Fig. 3a) and GAGs (Fig. 3b), while sinomenine-treated rats had considerably higher levels. Sinomenine (7.5 mg/kg) increased the levels of aggrecan and GAG.

### IFN-γ and MCP-1

**Figure 4:**
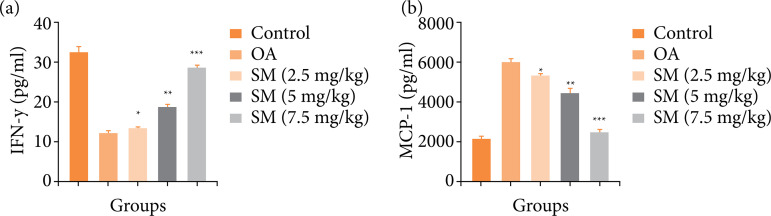
Effect of SM on the INF-γ and MCP-1 of MIA induced OA group rats. **(a)** INF-γ and **(b)** MCP-1. All the results are presented as mean ± SD (n=10 for each group) from three independent experiments.

OA: osteoarthritis; SM: sinomenine; IFN-γ: interferon gamma; MCP-1: monocyte chemoattractant protein-1; MIA: monosodium iodoacetate; SD: standard deviation; *P < 0.05; **P < 0.01; ***P < 0.001 vs. MIA-induced OA group; ^###^P < 0.001 vs. normal control.

OA group rats displayed the repressed level of IFN-γ (Fig. 4a) and enhanced level of MCP-1 (Fig. 4b) as compared to normal group rats. Sinomenine treated group rats significantly (P<0.001) improved the level of IFN-γ and reduced the level of MCP-1.

### Antioxidant parameters

**Figure 5:**
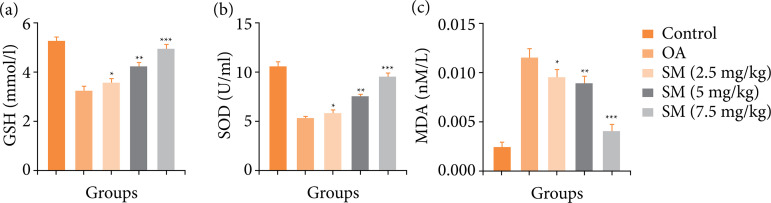
Effect of SM on the antioxidant parameters of MIA induced OA group rats. **(a)** GSH, **(b)** SOD and **(c)** MDA. All the results are presented as mean ± SD (n = 10 for each group) from three independent experiments.

OA: osteoarthritis; SM: sinomenine; GSH: glutathione; SOD: superoxide dismutase; MDA: malonaldehyde; MIA: monosodium iodoacetate; SD: standard deviation; *P < 0.05; **P < 0.01; ***P < 0.001 vs. MIA-induced OA group; ^###^P < 0.001 vs. normal control.:

Various diseases, such as osteoporosis, arthritis, cancer, and other ones, are accelerated by oxidative stress. The OA control group rats showed a similar result, with lower levels of GSH, SOD, and higher levels of MDA. Sinomenine treatment significantly (P<0.001) boosted the level of GSH (fig. 5a), SOD (fig. 5b) and downregulated the MDA level (fig. 5c).

### Inflammatory cytokines

**Figure 6:**
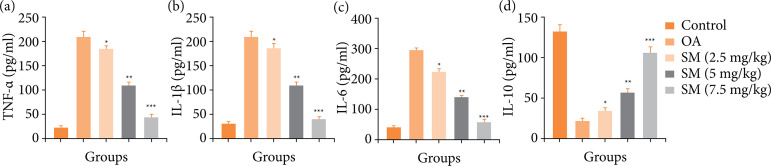
Effect of SM on the inflammatory cytokines of MIA induced OA group rats. **(a)** TNF-α, **(b)** IL-1β, **(c)** IL-6 and **(d)** IL-10. All the results are presented as mean ± SD (n = 10 for each group) from three independent experiments.

OA: osteoarthritis; SM: sinomenine; TNF: tumor necrosis factor; IL: interleukin; MIA: monosodium iodoacetate; SD: standard deviation; *P < 0.05; **P < 0.01; ***P < 0.001 vs. MIA-induced OA group; ^###^P < 0.001 vs. normal control.

The inflammatory reaction is important in the progression of OA disease. In this study, OA group rats displayed the augmented level of TNF-α (Fig. 6a), IL-1β (Fig. 6b), IL-6 (Fig. 6c) and suppressed the level of IL-10 (Fig. 6d). Sinomenine treated group rats significantly (P < 0.001) altered the level of inflammatory cytokines.

### Inflammatory parameters

**Figure 7:**
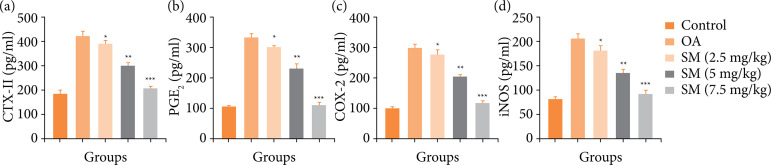
Effect of SM on the inflammatory parameters of MIA induced OA group rats. **(a)** CTX-II, **(b)** PGE_2_, **(c)** COX-2 and **(d)** iNOS. All the results are presented as mean ± SD (n = 10 for each group) from three independent experiments.

OA: osteoarthritis; SM: sinomenine; CTX-II: C-terminal cross-linked telopeptide of type II collagen; COX-2: cyclooxygenase-2; iNOS: inducible Nitric oxide synthase; MIA: monosodium iodoacetate; SD: standard deviation; *P < 0.05; **P < 0.01; ***P < 0.001 vs. MIA-induced OA group; ^###^P < 0.001 vs. normal control.

OA induced group rats displayed the boosted level of CTX-II (Fig. 7a), PGE_2_ (Fig. 7b), COX-2 (Fig. 7c) and iNOS (Fig. 7d). Sinomenine treated group rats significantly (P < 0.001) suppressed the level of inflammatory parameters. Sinomenine (7.5 mg/kg) treated group rats displayed the maximum suppression of inflammatory parameters and reached near to the normal control.

### Matrix metalloproteinases

**Figure 8:**
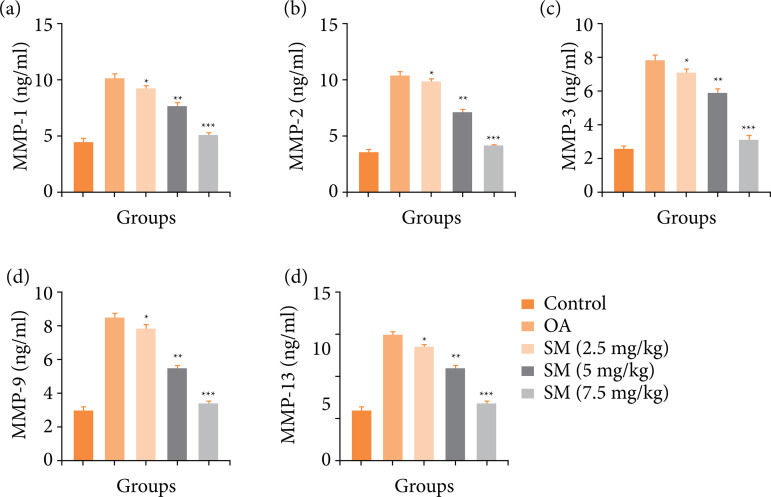
Effect of sinomenine on the MMPs parameters of MIA induced OA group rats. **(a)** MMP-1, **(b)** MMP-2, **(c)** MMP-3, **(d)** MMP-9 and **(e)** MMP-13. All the results are presented as mean ± SD (n = 10 for each group) from three independent experiments.

OA: osteoarthritis; SM: sinomenine; MMP: matrix metalloproteinase; MIA: monosodium iodoacetate; SD: standard deviation; *P < 0.05; **P < 0.01; ***P < 0.001 vs. MIA-induced OA group; ^###^P < 0.001 vs. normal control.

MMPs are important in the propagation and progression of OA disease. OA induced group rats displayed the boosted level of MMP-1 (Fig. 8a), MMP-2 (Fig. 8b), MMP-3 (Fig. 8c), MMP-9 (Fig. 8d), and MMP-13 (Fig. 8e). Sinomenine treatment remarkably repressed the level of MMPs. Sinomenine (7.5 mg/kg) treated group rats showed the maximum suppression in the level of MMPs.

## Discussion

The OA disease is categorized via progression of articular cartilage loss and the growth of bone osteophytes at the joint borders^25^. The available treatments for OA disease are surgical procedure, medical and non-medical therapy, which are more focus on the symptom therapies such as reducing pain and enhancing the overall function^26^. However, due to limitations, the researcher focuses their research to scrutinize the more protective drug from the natural source.

The reduction in the body weight is commonly observed during the OA disease. The body weight reduced due to loss or deformation of bone structures^2^^,^ 6. Rats in the OA control group had a similar result. No difference was observed in the spleen and kidney weight, but heart weight increased in the OA control group rats due to increase protein synthesis like IL-6, TNF, MMP-1 at the cellular level and Sinomenine treatment effectively suppressed the level of cytokines and MMP, which suggested the suppression of protein synthesis and reduced the heart weight.

Joint swelling is induced via synovial infiltration and inflammatory reaction, after the MIA injection^27^. Hyperplasia with fibrosis of the synovial membrane, resorption of the articular cartilage surface, and edema with inflammatory cell infiltration in the surrounding cartilage with congested blood vessels were all seen during the OA disease. This experimental investigation also found a similar effect, indicating that OA disease is progressing and spreading. OA rodent model either naturally, chemically or surgically widely accepted to scrutinize the cartilage degeneration pathology and potentially therapeutic alteration of disease^3^^,^ 28.

Previous report suggests that the chemically induced OA disease similarly occurs in human beings with age. Moreover, the OA disease is a long-time period chronic disease and it needs a long time to scrutinize the protective effect of tested drug in rodent. MIA induced OA group rats exhibited the prominent knee joint swelling due to increase the joint volume as compared to the normal group rats. The joint swelling reached maximum seven days and remained persistent at the end of the experimental study. In this experimental study, we selected the rodent model (MIA induced OA disease) to scrutinize the protective effect of tested drugs.

In this experimental study, we used the injection of MIA to induce the OA. The administration of MIA disrupts the metabolism of chondrocyte due the suppression of glycolysis and inducing the deterioration of cartilage^27^. The collapsing joint’s histology closely resembles that of a human joint, which is the model’s principal benefit^29^. Finally, the MIA exposed to the subchondral bone, injured the synovium, and induced the pain in the joint^27^. Due to induction of OA in the rodent similarly to the human, this model gets popularity day by day to scrutinize the therapeutic and preventive effect of tested drug.

It is well known that ROS induces the DNA injury. This injury may be attributed to enhanced the production of inflammatory cytokines^27^. The level of ROS boosted during the degradation cartilage injury, which was confirmed via estimation of lipid peroxidation in the both circulation and cartilage area^30^^,^ 31. The level of GSH suppressed during the OA disease due to crucial role of nitrosative and oxidative stress in the pathogenesis of OA disease^32^.

It is well known that matrix turnover depends on the chondrocytes, which starts the production of inflammatory cytokines in rodent, as well as human^33^. Several cytokines such as IL-1, IL-1β, TNF-α, IL-10, IL-17 and increased level of MMPs play a considerable role in the degradation of cartilage^34^. OA induced group rats exhibited the alter level of cytokines, which was modulated by the sinomenine.

It is well known that the degradation of cartilage starts during the OA disease, and it is considered as the biomarker of the disease^25^. The deterioration of cartilage begins during OA disease due to inductions of cartilage matrix products such as MMPs, which leads to collagen network dysfunction^35^^,^ 36. MMPs are the proteinases family member that can degrade the all-extracellular matrix components^34^. Gelatinases (type IV collagenases) are the member of MMP family and divided into different subclasses such as MMP-2 (gelatinase A) and MMP-9 (gelatinase B), which are responsible for the degrading types collagens IV, collagen V, gelatin and elastin^37^. MMP-2 is known to be generated via osteoblasts and tissue structural cells such as endothelial cells and fibroblasts. MMP-9 generated via inflammatory cells include eosinophils, macrophages, and neutrophils^38^^,^ 39. MMPs (MMP-2 and MMP-9) are latent precursors that can be triggered with restricted proteolysis.

During the OA decease, the level of MMP-9 increased, which reflected the inflammatory reaction in the joints and exhibited the positive correlation between the rapid destruction of hip joint and MMP^34^. Another biomarker of OA disease is CTX-II, widely used for the estimation the status of degradation of cartilage^3^. PGE_2_ is an enzyme commonly used to identify the suppression of the proteoglycan synthesis and boost the cartilage matrix degradation^40^^,^ 41. MMP-1, MMP-3, PGE_2_, and CTX-II levels were substantially higher in the OA group rats, indicating the development of cartilage network dysfunction, while sinomenine treatment significantly reduced the level, indicating cartilage protection.

## Conclusion

Our data showed that the sinomenine remarkably improved the body weight and reduced the weight of heart tissue. Sinomenine considerably altered the level of antioxidant enzymes and repressed the level of NO, MCP-1 and boosted the level of aggrecan, GAGs, IFN-γ. Sinomenine remarkably modulated the level of inflammatory cytokines, inflammatory mediators and MMPs. Consequently, sinomenine treatment can be proposed to be a protective anti-osteoarthritic drug. More molecular study must be done to identify the exact mechanism for the treatment of osteoarthritis.

## Data Availability

The data will be available upon request
